# An assessment of discriminatory power of office blood pressure measurements in predicting optimal ambulatory blood pressure control in people with type 2 diabetes

**DOI:** 10.11604/pamj.2014.19.231.2608

**Published:** 2014-10-31

**Authors:** Andre Pascal Kengne, Christelle Nong Libend, Anastase Dzudie, Alain Menanga, Mesmin Yefou Dehayem, Samuel Kingue, Eugene Sobngwi

**Affiliations:** 1 South African Medical Research Council & University of Cape Town, Cape Town, South Africa; 2 Diabetes and Endocrine service, Yaounde Central Hospital, Yaounde, Cameroon; 3 Service of Internal Medicine, General Hospital of Douala, Douala, & Buea faculty of Health Sciences, Buea, Cameroon; 4 Service of Medicine A, General Hospital of Yaounde, Yaounde, Cameroon

**Keywords:** Ambulatory blood pressure, office blood pressure, diabetes mellitus, prediction, sub-Saharan Africa, Cameroon

## Abstract

**Introduction:**

Ambulatory blood pressure (BP) measurements (ABPM) predict health outcomes better than office BP, and are recommended for assessing BP control, particularly in high-risk patients. We assessed the performance of office BP in predicting optimal ambulatory BP control in sub-Saharan Africans with type 2 diabetes (T2DM).

**Methods:**

Participants were a random sample of 51 T2DM patients (25 men) drug-treated for hypertension, receiving care in a referral diabetes clinic in Yaounde, Cameroon. A quality control group included 46 non-diabetic individuals with hypertension. Targets for BP control were systolic (and diastolic) BP.

**Results:**

Mean age of diabetic participants was 60 years (standard deviation: 10) and median duration of diabetes was 6 years (min-max: 0-29). Correlation coefficients between each office-based variable and the 24-h ABPM equivalent (diabetic vs. non-diabetic participants) were 0.571 and 0.601 for systolic (SBP), 0.520 and 0.539 for diastolic (DBP), 0.631 and 0.549 for pulse pressure (PP), and 0.522 and 0.583 for mean arterial pressure (MAP). The c-statistic for the prediction of optimal ambulatory control from office-BP in diabetic participants was 0.717 for SBP, 0.494 for DBP, 0.712 for PP, 0.582 for MAP, and 0.721 for either SBP + DBP or PP + MAP. Equivalents in diabetes-free participants were 0.805, 0.763, 0.695, 0.801 and 0.813.

**Conclusion:**

Office DBP was ineffective in discriminating optimal ambulatory BP control in diabetic patients, and did not improve predictions based on office SBP alone. Targeting ABPM to those T2DM patients who are already at optimal office-based SBP would likely be more cost effective in this setting.

## Introduction

Blood pressure (BP) is a major determinant of the risk of cardiovascular disease, the main killer in diabetes [1, 2]. There is abundant evidence on the effectiveness of blood pressure control in reducing the risk of macrovascular and microvascular disease in people with diabetes [3, 4]. However, achieving and maintaining optimal BP control is a very challenging commitment in this population. Accordingly, in many settings, less than a third of people with diabetes in the upper part of BP distribution, otherwise known as ‘with hypertension’ achieve adequate BP control.

Accurate BP measurement is a key component of strategies aiming to reduce blood pressure related risk. This involves approximating as much as possible the true current levels of BP, but also directing BP appraisal at those indices and measurements that are better correlated with future risk of BP related health hazards [5]. Numerous observational studies have demonstrated ambulatory BP monitoring (ABPM) to be superior to clinic measurements in predicting target organ damage and other clinical outcomes associated with higher-than-optimal blood pressure [6]. In both people with and without diabetes, ABPM has been traditionally recommended for a number of indications including diagnosis of white-coat hypertension, investigation of drug resistance, hypotensive symptoms, episodic hypertension, and autonomic dysfunction [7, 8]. Recently, routine use of ABPM has been recommended for initial diagnosis of hypertension in the general population in some settings [6, 9], but not acclaimed everywhere [10]. There is also a continuing debate about whether ABPM should be routinely used to diagnose hypertension and tailor hypertensive medication in every patient with diabetes [11–14]. In general, devices availability and cost of monitoring have been identified as limiting factors to the uptake of ABPM based strategies, including in affluent settings [10]. Accordingly, more targeted strategies are needed, but supporting evidences are still lacking, particularly in resources-limited setting.

The aim of this study was to assess the diagnostic capability of office BP measurements in predicting optimal ambulatory BP control in sub-Saharan African with type 2 diabetes mellitus. We draw comparisons of the observed effects with those in people without diabetes.

## Methods

### Study setting and participants

This cross-sectional study was conducted at the National Obesity Centre of the Yaounde Central Hospital in the Capital city of Cameroon. The study setting has been described in details previously [15, 16]. Participants with diabetes were enrolled on a consecutive basis during outpatient visits between July 2009 and February 2010. The study complies with the Declaration of Helsinki, was approved by the National Ethic Committee and informed consent obtained from each participant.

Participants were patients with type 2 diabetes and hypertension for whom BP control medications had not changed over the three preceding months. Were excluded from the study patients on night-time shift, patients with arrhythmia which precluded accurate BP measurement through oscillometric method, and patients with arm circumference greater than 32 centimetres and for whom larger cuff size was required. For quality control purpose, a group of 43 diabetes-free hypertensive adults was also recruited. They were all individuals who underwent ABPM at the Doula General Hospital for various purposes indications. Participants underwent clinical and paraclinical examinations with data collected on age, gender, diagnosed duration of diabetes, blood pressure variables and treatments, fasting blood glucose, haemoglobin A1c (HbA1c), lipid profile, serum urea and creatinin.

### Blood pressure measurement

Office BP measurement was performed by the same investigator with the use of a Spencer^®^ aneroid sphygmomanometer, following the Riva-Rocci Korotkoff method [17] in participants with diabetes, and with an automated sphygmomanometer (SPENGLER electronic Pro M) and appropriate cuff sizes (13×23 cm or 16×30 cm) in diabetes-free participants. Three consecutive measurements 5 minutes apart were conducted at rest in each subject in a seated position, and average of the last two measurements used in the study. Systolic and diastolic BP were based respectively on phase I and phase V of the Korotkoff sounds. Ambulatory BP was measured and recorded with a BOSO (BOSCH + SOHN GmbH & Co. KG, Germany) TM^®^ 2430 PC2 device in participants with diabetes, and with a DIASYS INTEGRA II (NOVACOR, FRANCE) device in diabetes-free participants. The BP cuff was displayed on the non-dominant arm in participants when the between-arm office-based difference systolic BP was less than 10 mmHg; otherwise the cuff was displayed on the arm with the highest office-based systolic BP. The two first measurements was recorded under the supervision, and subsequent measurements were recorded every 15 minutes between 7 a.m. and 10 p.m., and every 30 minutes between 10 p.m. and 7 a.m. The device was collected after 24 hours of recording and data transferred to a computer with the use of the Profil-Manager 3^®^ software (BOSCH + SOHN GmbH & Co. KG, Germany) for participants with diabetes, and with the HolterSoft Ultima version 2.4.4 (NOVACOR, FRANCE) in diabetes-free participants. Records were considered to be of good quality when they spanned the entire 24 hours, had at least 70% of valid measurements and no gap of more than 2 hours without a single measurement.

### Definitions

The following definitions were applied: 1) BP controlled based on Office measurements: systolic (and diastolic) BP < = 130 (80) mmHg on at least two consecutive visits over at least the three preceding months [18, 19]; 2) BP not controlled based on Office measurements: systolic (and/or diastolic) BP >130 (80) mmHg on at least two consecutive visits over at least the three preceding months; 3) BP controlled over 24h: mean 24h systolic (and diastolic) BP < = 130 (80) mmHg [20].

### Statistical methods

Data were analyzed with SAS/STAT version 9.1 (SAS Institute, Cary, NC). Data are summarised as mean (standard deviation, SD), median (minimum-maximum) or count (percentages). Group comparisons used Person χ2 test and equivalents for qualitative variables, and Student t-test and Mann-Whitney U test for quantitative variables. The concordance between different BP measurements was assessed with the Pearson correlation coefficient. Logistic regressions were used to assess the association between office BP variables and optimal BP control based on ABPM. The area under the receiver operating characteristic curve (AUC) then assessed and compared the ability of office BP variables to predict optimal control based on ABPM. AUC comparisons used non-parametric methods [21]. A p-value

## Results

### Baseline characteristics of participants

Of the 51 participants with type 2 diabetes included, 26 (51%) were women and 32 (63%) were at target office BP control Table 1. With the exception of office blood pressure variables and fasting glycaemia. The mean age of the diabetic cohort was 60.3 years (SD = 10.2) and they had been diagnosed with diabetes since a median duration of 6 years (min-max: 0-29). Participants without diabetes were comparable to those with diabetes with regard to many baseline characteristics. However, they were likely younger, had higher levels of office BP and serum creatinin (all p<0.001). In addition, more were receiving a beta blocker (23% vs. 6%, p=0.02), and fewer receiving an ACE inhibitor (51% vs. 82%, p=0.001) or a diuretic (56% vs. 78%, p=0.02; Table 1).

**Table 1:** Profile of 51 patients with type 2 (at or not at target clinical blood pressure control) and 43 non-diabetic participants

Variables	Diabetes				No diabetes	P-value
	Optimal control	Non optimal control	p	Overall		
N	32	19		51	43	
Sex, Men:Women	13:19	12:7	0.15	25:26	26:17	0.27
Mean age, years (SD)	60.0 (10.8)	60.9 (9.3)	0.77	60.3 (10.2)	49.3 (11.5)	<0.001
Median duration of diagnosed hypertension (min-max), years	4.0 (0-24)	5 (1-14)	0.45	5.0 (0-24)	3 (0-17)	0.25
Median duration of diagnosed diabetes (min-max), years	5 (0-29)	7 (1-26)	0.12	6 (0-29)	-	-
Mean body mass index, kg/m^2^ (SD)	27.5 (3.5)	28.1 (4.4)	0.63	27.7 (3.9)	28.3 (4.1)	0.42
Mean waist circumference, cm (SD)	99.0 (10.3)	97.3 (12.0)	0.59	98.4 (10.9)	-	-
Mean systolic BP, mmHg (SD)	118.6 (7.9)	137.2 (10.1)	<0.001	125.5 (12.5)	159.5 (24.8)	<0.001
Mean diastolic BP, mmHg (SD)	70.9 (7.1)	80.5 (10.1)	<0.001	74.5 (9.5)	93.2 (14.9)	<0.001
Median number of BP agents (min-max)	2 (1-4)	2 (1-3)	0.50	2 (1-4)	2 (0-5)	0.10
Angiotensin coverting enzyme inhibitors, n (%)	29 (91)	13 (68)	0.06	42 (82)	22 (51)	0.001
Diuretics, n (%)	26 (81)	14 (74)	0.72	40 (78)	24 (56)	0.02
Calcium chanel blockers, n (%)	13 (41)	10 (53)	0.40	23 (45)	21 (49)	0.72
Beta blockers, n (%)	3 (9)	0	0.28	3 (6)	10 ((23)	0.02
Angiotensin receptor II antagonists, n (%)	1 (3)	2 (10)	0.54	3 (6)	3 (7)	>0.99
Mean serum urea, g/L (SD)	0.34 (0.16)	0.34 (0.15)	0.94	0.34 (0.16)	0.34 (0.16)	0.39
Median serum creatinin, g/l (min-max),	9.7 (5-21)	9.6 (6-13)	0.74	9.6 (5-21)	11.0 (7.5-52.0)	<0.001
Median tryglicerides, g/l (min-max)	1.18 (0.29-1.18)	0.93 (0.55-1.51)	0.39	1.07 (0.29-2.73)	1.10 (0.49-1.85)	0.91
Mean fasting glycaemia, g/l (SD)	1.16 (0.36)	1.60 (1.02)	0.03	1.32 (0.71)	-	-
Mean HbA1c,% (SD)	7.59 (1.76)	8.31 (2.94)	0.29	7.85 (2.26)	-	-
Mean Total cholesterol, g/l (SD)	1.96 (0.31)	1.76 (0.40)	0.07	1.89 (0.35)	1.89 (0.36)	0.99
Mean HDL cholesterol, g/l (SD)	0.50 (0.28)	0.46 (0.14)	0.59	0.49 (0.23)	0.43 (0.12)	0.14
Mean LDL cholesterol, g/l (SD)	1.34 (0.38)	1.10 (0.44)	0.06	1.26 (0.41)	1.22 (0.31)	0.66

### Ambulatory blood pressure profile

Ambulatory blood pressure profile is depicted in Table 2 for participants with and without diabetes. As expected, for any given pressure variable, the daytime ABPM value was always higher than the night-time equivalent. The magnitude of the between-group difference in BP variable observed with office measurements substantially decreased when ABPM variables were considered. For instance there was no difference in pulse pressure between diabetic and non-diabetic participants based on daytime, night-time or 24-h ABPM values (all p > 30). Among participants with diabetes, there was no difference between those at target and those not at target level (based on office measurements) for all night-time ABPM variables (all p > 0.05, Table 2). In addition, daytime and 24-h ABPM DBP and MAP were not appreciably different between the two groups (all p > 0.08, Table 2).

**Table 2:** Blood pressure variables in participants with and without diabetes

Variables	Diabetes				No diabetes	P-value
	Optimal control	Non optimal control	p	Overall		
N	32	19		51	43	
**Office variables**						
SBP	118.6 (7.9)	137.2 (10.1)	<0.001	125.5 (12.5)	159.5 (24.8)	<0.001
DBP	70.9 (7.1)	80.5 (10.1)	<0.001	74.5 (9.5)	93.2 (14.9)	<0.001
PP	47.7 (8.5)	56.6 (13.7)	0.02	51.0 (11.5)	66.2 (16.9)	<0.001
MAP	86.8 (6.2)	99.4 (7.7)	<0.001	91.5 (9.1)	115.3 (17.0)	<0.001
**Daytime ABPM**						
SBP	135.4 (10.0)	143.5 (12.7)	0.01	138.4 (11.6)	147.8 (24.3)	0.02
DBP	82.0 (6.9)	84.0 (7.9)	0.33	82.7 (7.3)	90.4 (15.7)	0.005
PP	53.5 (7.2)	59.4 (8.5)	0.01	55.7 (8.1)	57.3 (12.1)	0.44
MAP	99.8 (7.3)	103.9 (8.9)	0.08	101.3 (8.1)	109.5 (18.1)	0.008
**Night-time ABPM**						
SBP	125.8 (12.2)	132.9 (19.1)	0.16	128.4 (15.3)	137.9 (24.5)	0.03
DBP	74.6 (8.6)	76.7 (8.6)	0.46	75.4 (9.5)	82.4 (15.6)	0.01
PP	51.2 (8.1)	56.2 (9.8)	0.05	53.0 (9.0)	55.5 (13.0)	0.30
MAP	91.7 (9.2)	95.4 (13.4)	0.24	93.1 (11.0)	100.9 (18.0)	0.02
**24-h ABPM**						
SBP	133.4 (9.6)	141.2 (13.4)	0.02	136.3 (11.7)	145.0 (23.7)	0.03
DBP	80.4 (6.5)	82.5 (8.0)	0.33	81.2 (7.1)	87.7 (15.2)	0.01
PP	53.0 (7.1)	58.7 (8.5)	0.01	55.1 (8.1)	57.3 (11.7)	0.30
MAP	98.1 (6.9)	102.1 (9.3)	0.09	99.6 (8.0)	106.8 (17.6)	0.02
**Min-Max**						
SBP min	91.6 (14.2)	93.4 (14.5)	0.66	92.3 (14.2)	104.3 (24.7)	0.006
DBP min	47.2 (5.4)	47.2 (5.4)	0.98	47.2 (5.7)	60.8 (15.6)	<0.001
SBP max	204.7 (20.7)	213.3 (27.4)	0.21	207.9 (23.5)	207.5 (39.7)	0.95
DBP max	143.3 (15.5)	142.7 (18.9)	0.90	143.1 (16.7)	133.2 (25.4)	0.03

### Correlation between BP variables

In people with diabetes, correlation coefficients were low-to-good among concomitant BP variables (Table 3). Among concomitant variables, the higher correlation coefficient was always recorded between DBP and MAP, and the lowest, and mostly non-significant between DBP and PP. The pattern was similar in participants without diabetes, with however mostly significant correlation between DBP and PP (Table 4). When the associations of office BP variables with the ABMP equivalents were examined, the highest point estimate of the correlation coefficient in people with diabetes was always recorded for PP, followed by SBP, then MAP and DBP with nearly similar point estimates. In non-diabetic participants, the pattern again, was mostly similar. However correlation coefficients were mostly the same SBP and PP with their daytime or night-time equivalents, and always lower for office DBP and equivalents.

**Table 3:** Correlation matrix for different BP variables in participants with diabetes

Measurements	Office measurements				Daytime measurements				Night-time measurements				24-h measurements			
	SBP	DBP	PP	MAP	SBP	DBP	PP	MAP	SBP	DBP	PP	MAP	SBP	DBP	PP	MAP
**Office**																
SBP	1	0.488	0.690	0.796	0.563	0.314	0.524	0.458	0.467	0.356	0.421		0.571	0.355	0.517	0.485
DBP		1	-0.296	0.917	0.289	0.522	-0.053	0.451	0.113	0.344	-0.168	0.251	0.260	0.520	-0.079	0.432
PP			1	0.110	0.376	-0.089	0.617	0.127	0.417	0.105	0.601	0.255	0.409	-0.044	0.631	0.173
MAP				1	0.458	0.506	0.203	0.522	0.292	0.401	0.076	0.367	0.441	0.523	0.182	0.522
**Daytime**																
SBP					1	0.719	0.785	0.909	0.717	0.547	0.648	0.650	0.982	0.736	0.779	0.909
DBP						1	0.134	0.943	0.417	0.585	0.096	0.531	0.680	0.974	0.133	0.902
PP							1	0.456	0.651	0.259	0.839	0.453	0.794	0.182	0.992	0.493
MAP								1	0.593	0.612	0.368	0.629	0.877	0.935	0.452	0.975
**Night-time**																
SBP									1	0.841	0.822	0.950	0.836	0.573	0.710	0.742
DBP										1	0.382	0.968	0.659	0.754	0.296	0.762
PP											1	0.603	0.732	0.185	0.900	0.464
MAP												1	0.769	0.701	0.501	0.785
**24 hours**																
SBP													1	0.736	0.805	0.918
DBP														1	0.191	0.944
PP															1	0.503
MAP																1

**Table 4:** Correlation matrix for different BP variables in non-diabetic participants

Measurements	Office measurements				Daytime measurements				Night-time measurements				24-h measurements			
	SBP	DBP	PP	MAP	SBP	DBP	PP	MAP	SBP	DBP	PP	MAP	SBP	DBP	PP	MAP
**Office**																
SB	1	0. 7	0.8	0.9	0.5	0.5	0.5	0.4	0.5	0.5	0.4	0.5	0.6	0.5	0.4	0.5
P		46	09	21	97	14	31	63	53	22	13	52	01	68	77	96
DBP		1	0.212	0.946	0.420	0.491	0.205	0.471	0.363	0.523	0.054	0.467	0.431	0.539	0.170	0.503
PP			1	0.517	0.505	0.320	0.597	0.411	0.489	0.304	0.555	0.397	0.502	0.357	0.549	0.430
MAP				1	0.535	0.536	0.378	0.549	0.482	0.560	0.233	0.542	0.543	0.591	0.331	0.583
**Daytime**																
SBP					1	0.904	0.833	0.969	0.874	0.797	0.687	0.856	0.987	0.894	0.832	0.957
DBP						1	0.516	0.592	0.730	0.838	0.366	0.815	0.884	0.951	0.550	0.943
PP							1	0.670	0.790	0.507	0.879	0.650	0.833	0.560	0.955	0.695
MAP								1	0.815	0.843	0.521	0.856	0.951	0.949	0.689	0.972
**Night-time**																
SBP									1	0.882	0.822	0.962	0.940	0.839	0.795	0.907
DBP										1	0.456	0.977	0.851	0.931	0.510	0.919
PP											1	0.636	0.746	0.459	0.885	0.601
MAP												1	0.917	0.918	0.655	0.942
**24 hours**																
SBP													1	0.909	0.840	0.971
DBP														1	0.537	0.982
PP															1	0.685
MAP																1

All r < = ∣0.304∣ are non-significant (p > = 0.05)

### Prediction of optimal ambulatory blood pressure control

**Figure 1:**
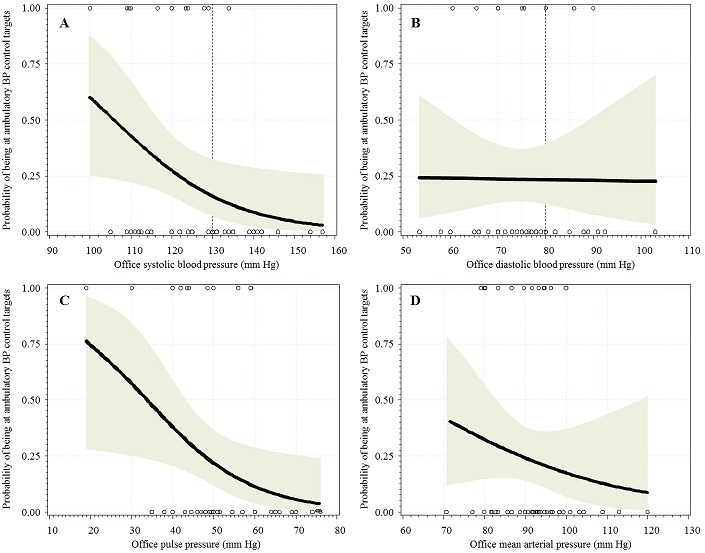
A) logistic curves showing the predicted and observed probability of being at target blood pressure (BP) levels based on ambulatory BP measurement and at different levels of office-based systolic blood pressure; B) diastolic blood pressure; C) pulse pressure; D) mean arterial pressure (in participants with diabetes)

**Figure 2:**
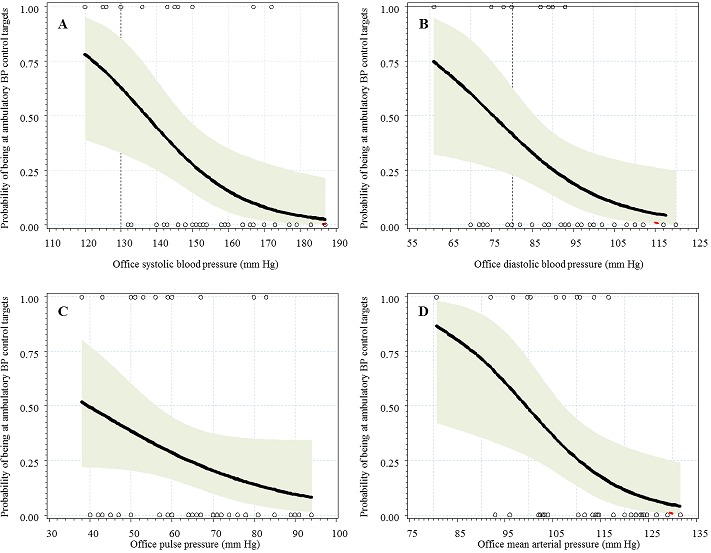
A) logistic curves showing the predicted and observed probability of being at target blood pressure (BP) levels based abulatory BP measurement and at different levels of office-based systolic blood pressure; B) diastolic blood pressure; C) pulse pressure; D) mean arterial pressure (in participants without diabetes)

Logistic regression curves are shown in Figure 1, Figure 2, illustrating the correlation of each office BP with optimal BP control based on ABPM measurements. In people with diabetes, there was no correlation between office DBP and optimal ambulatory control, while such a correlation was apparent for SBP, PP and MAP (in participants with diabetes), and for the 4 variables in non-diabetic participants. In people with diabetes, the AUC (95% confidence interval) for each office variable in predicting optimal ambulatory control was 0.717 (0.564-0.870) for SBP, 0.712 (0.542-0.880) for PP, 0.502 (0.314-0.690) for DBP and 0.583 (0.396-0.771) for MAP. Adding DBP to SBP, or MAP to PP had little effect (AUC =0.719 (0.558’ > AUC =0.719 (0.558-0.880) for each of the combinations). Difference in AUC was significant when comparing SBP with DBP (p = 0.037), but not for other comparison (?2 = 6.8, p = 0.15, df = 4 for all AUC comparisons). In non-diabetic participants (Figure 2), AUCs were 0.805 (0.644-0.967) for SBP, 0.801 (0.665-0.937) for MAP, 0.763 (0.623-0.902) for DBP, 0.695 (0.511-0.878) for PP and 0.813 (0.667-0.958) for SBP + DBP or PP + MAP. Again, SBP was better than DBP (p = 0.034 for AUC comparison), but all other comparisons were not statistically significant (?2 = 8.05, p = 0.09, df = 4 for all AUC comparisons). There was no evidence of a threshold of SBP or DBP below which optimal ambulatory control could be predicted with certainty. However, diabetic patients at optimal ambulatory control were likely to have office SBP Figure 1). However, such comparisons were based on few participants.

## Discussion

In this group of type 2 diabetic patients with hypertension and acceptable-to-good blood pressure control based office measurement, the 24-hour ambulatory blood pressure control was rather poor-to-acceptable. This was primarily driven by less optimal ambulatory pressure control during daytime opposed to night-time. Among concomitant office or ambulatory pressure variables, SBP was always strongly correlated with other variables than DBP, while between office variables and their ambulatory equivalents, the strongest correlation was always observed with pulse pressure and to some extend SBP. Based on AUC comparisons, office SBP outperformed DBP in predicting optimal 24-hour ambulatory control. All patients not at optimal office SPB were also likely not to be at optimal 24-hour control. Comparisons with non-diabetic participants suggested that some of these findings were likely specific to people with diabetes.

The agreement between office and ambulatory BP measurements has been examined in few previous studies in people with diabetes [22–24]. Correlations studies have been consistent in reporting only low-to-average correlation between office SBP, DBP and their ambulatory equivalents [22–25]. The focus of previous studies on SBP and DBP offers less opportunity for comparison of our findings of largest correlation estimate always for office pulse pressure with its ambulatory equivalents. This finding was in keeping with less variation in the mean values of pulse pressure between office and ambulatory measurements. The modest correlation between office and ambulatory BP variables largely accounts for the discrepancy between the two set of measurement in gauging optimal control in many studies [22, 24, 25].

There have been recent attempts in people with diabetes to derive models for estimating ambulatory pressure variables from their office equivalents [24]. The modest performance of such models suggests that office variables which are usually single measurement cannot reliably predict their ambulatory equivalents, the average of several measurements. Our findings even suggested that office DBP may be less useful in predicting optimal ambulatory control based on both SBP and DBP. There is already substantial evidence that DBP is less useful that SBP or PP in predicting long term cardiovascular outcome in people with diabetes [5]. In spite of the only modest-to-acceptable capacity of office BP variable to predict ambulatory control in our study, an encouraging observation was the fact that office variables, and SBP in particular were good at ruling out non-optimal ambulatory control. This has interest in the sense that if confirmed, it would help to direct ambulatory BP measurement for the purpose of treatment monitoring only to those people with diabetes who have already achieved stable optimal office BP control. Investigators in other settings have also suggested that ambulatory BP monitory may not be needed in all people with diabetes [24]. Our study has some limitations. This includes the small sample size and accordingly our limited power to reliably perform subgroup analysis. The absence of standardisation of the inclusion criteria across diabetic and non-diabetic subgroups could potentially bias some of our comparisons. For instance, some low and non-significant correlation in people with diabetes would be accounted for at least in part by the narrow range of BP measurement in people with diabetes compared to their non-diabetic counterparts. Definition of optimal ambulatory control was based on cut-off not validated in people with diabetes. In the absence of such cut-offs, studies in people with diabetes have generally relied on cut-off applicable to non-diabetics [4, 15, 21]. Having to recruit participants with diabetes and a non-diabetic quality control group in this resources-limited setting, cost implications obliged the recruitment of the non-diabetic cohort in a different health facility where ABPM was selectively applied in patients with hypertension on a routine basis. That two slightly different protocols for measuring BP (different devices) were applied across the two health facilities could potentially affect some of our comparisons. Our study also has major strengths including the use of a comparative non-diabetic sub-cohort, our ability to assess several common BP variables, to assess the capacity of office variables to predict optimal ambulatory control across the continuum of office variables, and the opportunity of comparing the performance of different office variables.

## Conclusion

Evidences from clinical trials have established the safety and efficacy of blood pressure lowering in people with diabetes, regardless of the starting blood pressure levels, in reducing cardiovascular risk [4]. Its remain however important in those at higher risk and will derive the most benefit from blood pressure lowering, to appropriately tailor the intensity of treatment in order to achieve the dual goal of optimising pressure control while minimizing risks from excessive BP lowering. This would be the case in people with diabetes, particularly those with longstanding disease who are already at greater risk of orthostatic hypotension resulting from autonomic neuropathy [26]. Our study suggests that ambulatory measurements, a more reliable approach for approximating the true pressure levels, would be useful for appraising blood pressure control only in those individuals with diabetes who are already at recommended targets of office blood pressure control. This has relevance in resources-poor setting where device availability and cost of monitoring are severe limiting factors to the uptake of ambulatory blood pressure measurement approaches. However, our finding would need to be confirmed and refined in larger studies.
